# Maternal Germinal Trisomy 21 in Down Syndrome

**DOI:** 10.3390/jcm3010167

**Published:** 2014-01-28

**Authors:** Maj A. Hultén, Linn Öijerstedt, Erik Iwarsson, Jon Jonasson

**Affiliations:** 1 Department of Molecular Medicine and Surgery, Karolinska Institutet, Karolinska University Hospital, Stockholm S-171 76, Sweden; E-Mail: erik.iwarsson@ki.se; 2 Department of Neurobiology, Care Sciences and Society, KI Alzheimer Disease Research Center, Karolinska University Hospital, Huddinge, Stockholm S-141 86, Sweden; E-Mail: linnstedt@gmail.com; 3 Department of Clinical Genetics, Karolinska University Hospital, Stockholm S-171 76, Sweden; 4 Department of Clinical and Experimental Medicine, Linköping University, LMC, University Hospital, Linköping S-581 85, Sweden; E-Mail: jon.jonasson@lio.se

**Keywords:** aneuploidy, Down syndrome, germinal mosaicism, maternal origin, trisomy 21

## Abstract

It has now been over 50 years since it was discovered that Down syndrome is caused by an extra chromosome 21, *i.e.*, trisomy 21. In the interim, it has become clear that in the majority of cases, the extra chromosome is inherited from the mother, and there is, in this respect, a strong maternal age effect. Numerous investigations have been devoted to clarifying the underlying mechanism, most recently suggesting that this situation is exceedingly complex, involving both biological and environmental factors. On the other hand, it has also been proposed that germinal trisomy 21 mosaicism, arising during the very early stages of maternal oogenesis with accumulation of trisomy 21 germ cells during subsequent development, may be the main predisposing factor. We present data here on the incidence of trisomy 21 mosaicism in a cohort of normal fetal ovarian samples, indicating that an accumulation of trisomy 21 germ cells does indeed take place during fetal oogenesis, *i.e.*, from the first to the second trimester of pregnancy. We presume that this accumulation of trisomy 21 (T21) cells is caused by their delay in maturation and lagging behind the normal cells. We further presume that this trend continues during the third trimester of pregnancy and postnatally, up until ovulation, thereby explaining the maternal age effect in Down syndrome.

## 1. Introduction

It is well known that aneuploidy is common in humans, leading to reproductive failure, intrauterine deaths and live born offspring affected by congenital defects and learning disabilities. We have focused our attention on trisomy 21 Down syndrome, this being the most common aneuploidy condition in the live born human population. It is clear that the majority of trisomy 21 (T21) conceptions are caused by a segregation error in maternal oocytes with an increasing risk dependent on maternal age. However, in spite of numerous investigations to this effect, the exact mechanism underlying this meiotic segregation error has not been clarified. Instead, it has become generally accepted that many different biological and environmental factors may be involved in giving rise to aneuploidy (reviewed in [1,2,3,4,5]). In contrast, we have recently proposed that in any one woman, the most likely precursor is the degree of T21 mosaicism that originally existed in her ovaries during her own fetal development [6]. In other words, we advocate that the crucial predisposing factor may be the proportion of immature oocytes with three rather than two chromosomes 21 that are available in the ovarian cortex to undergo the first meiotic division, taking place just before ovulation. Following on from Zheng and Byers [7], we have further proposed that the so-called maternal age effect is due to an accumulation of T21 oocytes during prenatal and postnatal development [8]. In other words, we advocate that the crucial predisposing factor may be the age-related proportion of oocytes with three rather than two chromosomes 21 that are available in the ovarian cortex to undergo the first meiotic division, taking place just before ovulation. The net effect of these two mechanisms is that even a low percentage of trisomic cells present in gonadal tissue or germinal cells significantly increases the risk of aneuploidy in the offspring [9].

In a previous study, using fluorescence *in situ* hybridization (FISH), we identified the occurrence of T21 germ cells in eight fetal ovaries, obtained following social termination of pregnancy (TOP) in the second trimester [6]. This study has now been extended to include 12 additional fetal ovaries, where TOP was performed during the first trimester of pregnancy. We show that in these cases, where TOP has taken place at an earlier gestational age, the incidence of T21 mosaicism is significantly lower. Thus, in accordance with our hypothesis, there is an apparent accumulation in the degree of T21 mosaicism during normal fetal oogenesis.

## 2. Results

We have used FISH with two chromosome 21-specific probes, labelled in different colors (red and green) in order to assess the incidence of T21 in fetal ovaries obtained following TOP for a social reason at the clinical gestational age of 9–11 weeks.

**Figure 1:**
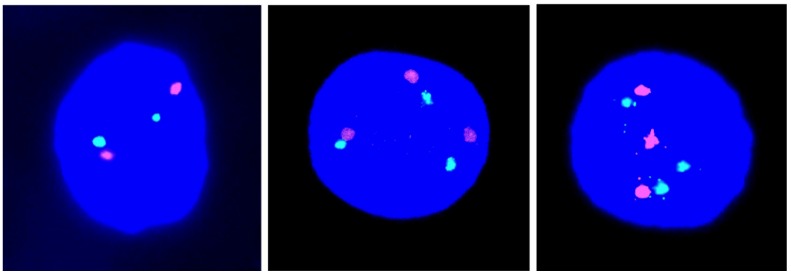
Illustrations of cell nuclei containing a different chromosome 21 copy number: Two red-green signals indicative of disomy 21 (left) and three red-green signals indicative of trisomy 21 (middle and right).

In this new series, we have recorded the incidence of T21 cell nuclei in 12 fetal ovarian samples, obtained during the first trimester of pregnancy. Examples of cell nuclei with T21 in comparison to the normal diploid are given in Figure 1. We identified a mean number of only 0.066% cell nuclei, showing T21 with a range of 0.00%–0.14% and an standard deviation (SD) of 0.045% in a total cell population of 27,042 (Table 1). This result is highly statistically significantly different (*p* < 0.0001) from the results (average, 0.54%; range, 0.20%–0.88%; SD, 0.23) obtained in our previous investigation of eight cases, ascertained after TOP in the second trimester (Figure 2 in [6]).

**Table 1:** Chromosome 21 copy number by fluorescence *in situ* hybridization (FISH) analysis of fetal ovarian cell nuclei. T21, trisomy 21.

ID Number	Gestational Age (weeks)	Number of Signals Red/Green *					Number of Analyzed Cells
		2 Red/2 Green	3 Red/3 Green	1 Red/1 Green	1 Red/2 Green	3 Red/2 Green	
		Normal	T21				
1	9	1998	2	0	0	0	2000
2	9	1994	1	1	2	2	2000
3	9	1988	2	3	2	5	2000
4	9	2000	2	0	2	1	2005
5	10	2934	4	8	7	7	2960
6	10	1997	2	1	0	0	2000
7	10	2100	0	0	0	0	2100
8	10	2394	2	1	2	1	2400
9	10	2495	1	2	0	2	2500
10	11	2148	2	0	0	0	2150
11	11	2995	0	1	0	4	3000
12	11	1999	0	1	0	0	2000
Total		27,042	18	18	15	22	27,115
Mean	9.9	99.73%	0.066%	0.066%	0.055%	0.081%	
SD	0.8	0.24%	0.045%	0.060%	0.071%	0.087%	

* The cell nuclei scored as having 2 red/2 green have been recorded as likely to be normal disomic, but may include some nuclei having 3 green or 1 green, considered most likely to be false positive or false negative green.

The cell nuclei scored as having a 1 red/1 green signal were recorded as either false negative monosomy 21 (due to the pairing of the two chromosomes 21) or true monosomy 21 [10,11] and those with 1 red/2 green and 3 red/2 green as likely false negative or false positive signals.

It should be added that in obtaining the estimate of T21 mosaicism, we have excluded cell nuclei showing deviations from the ones with three red together with three green signals, as these might be artifactual (Table 1). We also used a complementary chromosome 18-specific probe to differentiate between true T21 cell nuclei in relation to the potential occurrence of dual trisomy/triploidy. No such nuclei were identified in this series of fetal ovarian cells.

## 3. Discussion

Ideally, any hypothesis on the origin of trisomy 21 Down syndrome should address and seek to explain the following issues.

### 3.1. The Preponderance of Maternal Origin

We suggest that the reason for the preponderance of the maternal origin is the substantial sex difference in degree of T21 germinal mosaicism with a much higher incidence in fetal ovaries than testes [12]. To our knowledge, there are no explicit hypotheses in the literature, as regards this question, other than the suggestion that maternal meiotic chromosome mal-segregation is more common than paternal and that the mechanisms of origin may therefore be different.

### 3.2. The Changes in Maternal Recombination Patterns

In our view, the changes in maternal recombination patterns, as seen by family linkage analysis, are most readily explained by the expected patterns in a T21 oocyte in relation to that in a normal disomy oocyte [6,8]. These maternal recombination patterns are firmly laid down in her fetal oocytes and cannot be altered by any factors later during development. This also includes the meiotic divisions, taking place just before ovulation and after fertilization. In our view, it is therefore unlikely that any factors, such as cohesion deficiency (further discussed below), may play a causative role in the mal-segregation process.

### 3.3. The Increased Recurrence Risk in Younger Women

We have suggested that the increased recurrence risk in younger women is likely to be caused by a higher incidence of fetal oogonial/oocyte T21 mosaicism [6]; reviewed in [9]. It has been previously documented that such women may show somatic T21 mosaicism, as well [13]. On the basis of cytogenetic/molecular data and also maternal and grandmaternal ages in Down syndrome families, Kovaleva [14] suggests that the normal grandmothers were older and proposed that they conceived offspring that were trisomic, but these conceptions subsequently became mosaics by so-called post-zygotic rescue. To date, it is not clear, however, to what extent such generalized trisomy 21, involving both germinal and somatic cell populations, may, in fact, explain an increased recurrence risk in younger mothers.

### 3.4. The Maternal Age Effect

**Figure 2:**
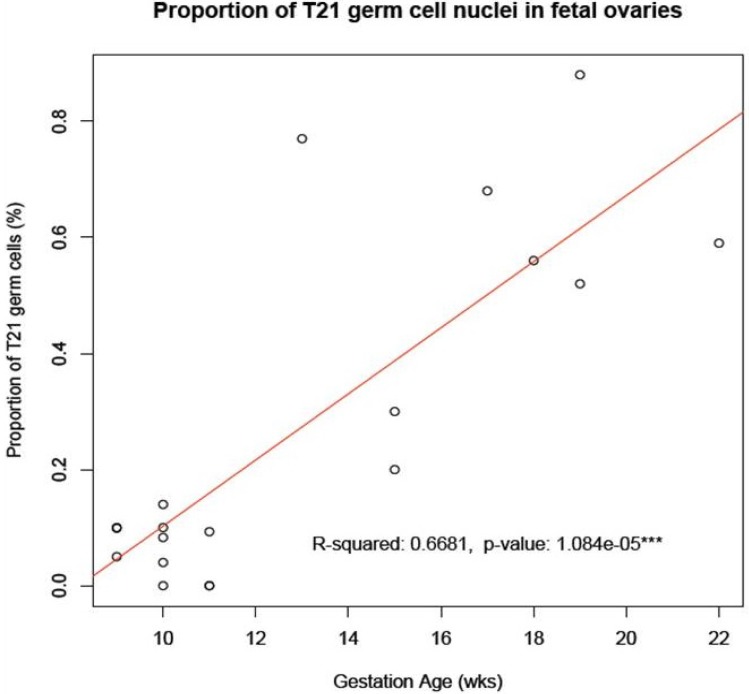
Accumulation of T21 oogonia/oocytes during fetal oogenesis. The graph shows the mean number of T21 oogonia/oocytes scored in samples of fetal ovaries during the first trimester (Table 1) in comparison to those during the second trimester (Table 1 in [6]). Note the apparent accumulation in incidence of T21 germ cells during the progression of oogenesis from the first to the second trimester of pregnancy.

In order to explain the so-called maternal age effect, we have proposed that there is an accumulation of T21 cells at prenatal and postnatal oocyte development, leading to a higher proportion at later maternal ages [8]. We have here shown that such an accumulation does indeed occur from the first to the second trimester of pregnancy in a sample of ovaries from fetuses with a normal mitotic karyotype (Figure 2). It remains to be investigated whether or not any additional accumulation may take place during postnatal development. As of yet, we have not been able to obtain the relevant cellular samples for this type of study, *i.e.*, primary oocytes in ovaries from women at different biological ages.

The occurrence of maternal germinal mosaicism as a predisposing factor has previously been implicated in a number of T21 (and other aneuploidy) cases through studies of oocytes and polar bodies, obtained for research in connection with *in vitro* fertilization (IVF) treatment [9,15]. It is noteworthy, though, that recent studies on human oocytes, retrieved in relation to IVF treatment, indicate that the types and frequency of maternal meiotic segregation errors differ substantially from those occurring in natural conception [2].

Much of the work, aiming to explain the mechanism(s) underlying the origin of aneuploidy with special reference to T21 Down syndrome, has been performed on other mammalian species, in particular using mouse models (reviewed in [16,17]). Special attention has been paid to the possibility that the maternal age effect is caused by an age-related deficiency in the cohesion complex, normally holding chromatids of the meiotic bivalents together until the first meiotic anaphase (see, e.g., [18,19]). No such age-related cohesion deficiency has, however, been noticed in the only study performed so far on human oocytes [20].

## 4. Materials and Methods

Human fetal ovarian samples were obtained from the Medical Research Council (MRC)/Wellcome Trust funded Human Developmental Biology Resource (HDBR, London, UK) with appropriate maternal written consent and approval from the local National Health Service (NHS) Health Authority Ethics Committee.

The samples were transported from the clinic to the HDBR resource and were staged, dissected and the tissue frozen on dry ice and stored at −80 °C following a minimum time delay, usually within 2 h of the termination of pregnancy. The age of the fetal samples was estimated following the staging guidelines of Hern [21]. A snip of tissue from the sample was taken prior to freezing to perform cytogenetic analysis to determine the mitotic karyotype. All the samples had a normal female karyotype (including Case 11 with the variant 46,XX,inv(9)(p11q12)).

The frozen samples were transported to our laboratory on dry ice and stored at −80 °C until analysis. Microscopy slides were prepared according to the technology described by Papadogiannakis *et al*. [22].

Microscopy slides for FISH analysis were fixed in 70% ethanol for 30 min and treated with pepsin (0.1 mg/mL) in 0.01 M HCl for 2 min at 37 °C. After additional washing in phosphate-buffered saline (PBS), paraformaldehyde (1%) fixation and dehydration through a series of alcohol, the slides were left to air-dry at room temperature.

Hybridization was performed according to the manufacturer’s instructions with two DNA probes positioned near the end of the long arm of chromosome 21 and labelled in SpectrumOrange and SpectrumGreen, respectively (Catalogue No. 32-190002, Abbot Molecular Inc., Des Plaines, IL, USA, and Cytocell, Catalogue No. LPT21QG/R, Cytocell Technologies Ltd., Cambridge, UK). A chromosome 18 centromeric probe labelled in SpectrumAqua was added to be able to differentiate between trisomy and triploidy (Catalogue No. 32-131018, CEP 18 (D18Z1) SpectrumAqua Probe). The DNA probes were mixed and added to the slides followed by denaturation, hybridization and post-hybridization washing. After dehydration, slides were mounted in glycerol containing 2.3% DABCO (1,4-diazabicyclo-(2,2,2) octane), as antifade and DAPI (4,6-diamino-2-phenyl-indole) 0.5 mg/mL for nuclear counterstaining. The cells selected for scoring were defined by the morphology of the nuclei, those being the only ones having large round or roundish nuclei [23].

Microscopy analysis was performed on a Zeiss Axioplan 2 microscope. Large cell nuclei, isolated from each other, were initially screened using the spectrum red light filter (Rhodamine). Images were captured and processed using the computer program, AxioVision. If an abnormal number of signals were detected using the red light filter, the spectrum green light filter (FITC) was switched on. Cell nuclei showing a dual red plus a green signal were scored as normal disomy 21, while those showing three dual signals were scored as T21, provided that the cell did not contain three chromosome 18 signals, which could be due to dual trisomy/triploidy. Any other combination of signals was considered likely to be artifactual and excluded. A minimum of 2000 cells per slide was analyzed.

## 5. Conclusions

In conclusion, it may seem unlikely that we will get to grips with the true origin of T21 Down syndrome, including, in particular, the maternal age effect, until such time as direct investigation on primary oocytes in the ovarian cortex may be performed. We would suggest that one possible way to obtain further information in this regard would be to record the incidence of T21 mosaicism in primary oocytes of the ovarian cortex, following oophorectomy in women at different biological ages. According to our own hypothesis [8], we would then expect to find an accumulation of T21 oocytes related to the increasing biological age of these women. Finally, it should be added that Rowsey *et al*. [24] stress that the type of technology that we have used for the counts of chromosome copy number is not applicable to cells at the pachytene stage of meiosis when homologues are synapsed. Further work is required to resolve this question.
